# Supplementary material for: Interplay between Hormones and Several Abiotic Stress Conditions on Arabidopsis thaliana Primary Root Development
Source: Cells. 2020 Dec 1;9(12):2576. doi: 10.3390/cells9122576 (PMC7759812; doi:10.3390/cells9122576)
Supplement: Supplementary file 1 [file cells-09-02576-s001.pdf]

Table S1. Proteins encoded by genes whose loss or gain of function were used in this review

HORMONE	SYNTHESIS		SIGNALING		TRANSPORT		CATABOLISM		REFERENCE
**AUXIN**	TAA1	TRYPTOPHAN AMINOTRANSFERASE OF ARABIDOPSIS1	TIR1	TRANSPORT INHIBITOR RESPONSE 1	PIN	PIN-FORMED			[13,30,256,266–270]
	YUC	YUCCA	AFB	AUXIN SIGNALING F-BOX PROTEIN	AUX1/ LAXS	AUXIN TRANSPORTER 1 /LIKE-AUXS			
	ASA1	ANTHRANILATE SYNTHASE alpha SUBUNIT 1	AUX/IAA	AUXIN/INDOLE ACETIC ACID	PILS	PIN-LIKES			
			ARFs	AUXIN RESPONSE					
			IAR	INDOLE-3-ACETIC ACID (IAA)-ALA HYDROLASE					
**CK**	IPT	ISOPENTENYL TRANSFERASE	AHK	ARABIDOPSIS HISTIDINE-KINASE RECEPTORS	**CONJUGATION**		CKX	CYTOKININ OXIDASES/ DEHYDROGENASES	[271–275]
			CRE1/ AHK4	CYTOKININ RESPONSE 1	UGT76C2 CYTOKININ GLYCOSYLTRANSFERASE				
			B-ARR	TYPE-B ARABIDOPSIS RESPONSE REGULATORS					
			A-ARR	TYPE-A-ARABIDOPSIS RESPONSE REGULATORS					
**GA**	GA1/ CPS	ENT-COPALYL DIPHOSPHATE SYNTHASE	SLY	SLEEPY					[276–278]
			DELLAs	GA INSENSITIVE (GAI)					
				REPRESSOR OF GAI (RGA)					
				RGA-LIKE (RGL)					
**BR**	DET2	DEETIOLATED2	BRI1	BRASSINOSTEROID INSENSITIVE1					[279–283]
			BRL1/3	BRASSINOSTEROID INSENSITIVE1-LIKE RECEPTOR KINASE 1/3					
			BSK	BRASSINOSTEROID-SIGNALING KINASE					
			BES1	BRI1-EMS-SUPPRESSOR1					
			BKI 1	BRASSINOSTEROID KINASE INHIBITOR 1					
			BZR1/2	BRASSINAZOLE RESISTANT 1/2					
			BIN2	BRASSINOSTEROID INSENSITIVE 2					
			BIL1/2	BIN2 like1/2					
**ABA**	ABA1 /ZEP	ZEAXANTHIN EPOXIDASE	ABI1	ABA INSENSITIVE 1					[284–287]
	ABA2 /SDR	SHORT-CHAIN DEHYDROGENASE/REDUCTASE							

Table S1. Proteins encoded by genes whose loss or gain of function were used in this review (continuation)

HORMONE	SYNTHESIS		SIGNALING		REFERENCE
**ABA**	ABA3/MCS	MOLYBDENUM COFACTOR SULFURASE	HAB1	HYPERSENSITIVE TO ABA1	
			HAI1/3	HIGHLY ABA-INDUCED1/3	
			AIP1 (HAI2)	AKT1-INTERACTING PHOSPHATASE1	
			PERK4	PROLINE-RICH EXTENSIN-LIKE RECEPTOR KINASE 4	
**ETHYLENE**	ETO1/HSP3	ETHYLENE OVERPRODUCER 1/ HYPERSENSITIVE TO PHOSPHATE STARVATION 3	ETR1/2	ETHYLENE RESPONSE 1/2	[288–291]
	ETO2/ACS	1-AMINOCYCLOPROPANE-1-CARBOXYLATE (ACC) SYNTHASE	EIN4	ETHYLENE INSENSITIVE 4	
			EBF1	EIN3-BINDING F BOX PROTEIN 1	
			EIN2/5/6	ETHYLENE INSENSITIVE2/5/6	
			CTR1	CONSTITUTIVE RESPONSE 1	
			EIN3/EIL 1	ETHYLENE INSENSITIVE3/EIN3-LIKE	
			ERF1	ETHYLENE RESPONSE FACTOR1	
**JA**	AOS	ALLENE OXIDE SYNTHASE	COI1/2	CORONATINE INSENSITIVE 1/2	[292–296]
	OPR3	12-OXOPHYTODIENOATE REDUCTASE 3	JAZ	JASMONATE ZIM DOMAIN PROTEINS	
			NINJA	NOVEL INTERACTOR OF JAZ	
			MYC2/3/4	Helix- loop-helix (bHLH) proteins	
**SA**	SID2	SALICYLIC ACID INDUCTION DEFICIENT 2 (ISOCHORISMATE SYNTHASE)	NPR1/3/4	NONEXPRESSOR OF PR GENES 1/3/4	[24,297,298]
**SL**	MAX1	CYTOCHROME P450 MONOOXYGENASE	MAX2	MORE AXILLARY GROWTH 1	[299,300]
	MAX3/CCD 7	CAROTENOID CLEAVAGE DIOXYGENASE 7			
	MAX3/CCD 8	CAROTENOID CLEAVAGE DIOXYGENASE 8			

Table S2. Primary root (PR) phenotype of loss or gain of function mutants of genes that participate in hormone homeostasis compared to WT under different abiotic stresses

Hormone	Mutant name	Mutant	Type of abiotic stress									Reference
			Osmotic	Salinity	ABA application	Cold	Oxidative	Heavy metal	Nutrient deficiency	High temperature	Alkalinity	
AUXIN	*yucID*	*GoF*						Strong inhibition in response to Al stress				[175]
	YUC1	*OE*							Short PR in response to low Pi			[221]
	YUC4	*OE*			Short							[144]
	yuc9	*LoF*						Long PR under Al stress.				[178]
	yuc8 yuc9	*LoF*						Long PR under Al stress				[93,178]
	wei2 (asa1)	*LoF*									Less affected	[265]
	taa1-1 (ckrc1-1, wei8)	*LoF*						Long PR under Al stress		Short	Less affected	[175,176,260,265]
	tir1-1	*LoF*			Long			Long under Cd treatment				[67,75,142,182]
	TIR1	*OE*							Short PR under high P			[222]
	tir1 afb2	*LoF*		Long			Long					[124]
	tir1-1 afb2-3	*LoF*								Short		[258]
	tir1 afb3	*LoF*					Long					[124]
	afb3-1	*LoF*							Not inhibited by NO3			[208]
	tir1 afb2 afb3	*LoF*							Short PR under high Pi			[222]
	iaa5-1	*LoF*	Short									[120]
	iaa7 (axr2-1)	*GoF*			Long				Long PR under high Pi			[67,75,142,217]
	iaa6-1	*LoF*	Short									[120]
	iaa14 (slr-1)	*GoF*						Long PR under Al stress. Long PR under Cr(VI) stress				[175,176,198]
	iaa17 (axr3)	*GoF*		Inhibited				Long PR under Cd or Cr(VI) stress. More sensitive to La3+				[93,182,194]
	iaa19-1	*LoF*	Short									[120]
	iaa28-1	*GoF*							Short PR under low or high Pi treatment			[217]
	arf1	*LoF*						Long PR under Al stress.				[175]

Table S2. Primary root (PR) phenotype of loss or gain of function mutants of genes that participate in hormone homeostasis compared to WT under different abiotic stresses (continuation)

Hormone	Mutant name	Mutant	Type of abiotic stress									References
			Osmotic	Salinity	ABA application	Cold	Oxidative	Heavy metal	Nutrient deficiency	High temperature	Alkalinity	
AUXIN	*arf2-101*	*LoF*			Hypersensitive to ABA, short PR							[143]
	ARF2	*OE*			Long							[143]
	arf6	*LoF*						Long PR under Al stress.				[175]
	arf8	*LoF*										
	arf9	*LoF*										
	arf10	*LoF*										
	arf16	*LoF*										
	arf7 arf19	*LoF*										
	arf10 arf16	*LoF*										
	pin1	*LoF*						More inhibition by As(III). Long PR under Cu excess. Less sensitive to La3+				[123,194,199]
	pin2 (eir1-1)	*LoF*		Short	Less sensitive to low ABA concentrations			More inhibition by As(III). Long PR under Al or Cd stress.	Long PR under ammonium or high Pi conditions. Short PR under low-B. Decreased growth under Fe stress	Short	Short	[66,67,75,121,142,174,175,182,194,217,264,265]
	pin2 (eir1-4)	*LoF*						More inhibition by As(III)				[174]
	PIN2	*OE*						Improved tolerance to As(III).				[174]
	pin4-3	*LoF*						Less sensitive to La3+				[194]
	pin1 pin3 pin7	*LoF*						Long PR under Cd stress				[182]
	pil6	*LoF*								Short		[257]
	PIL6	*OE*								Long		[257]
	PIN8	*OE*						Strong inhibition in response to Al stress				[175]
	aux1-7	*LoF*	Short	Short	Long			More inhibition by As(III). Long PR under Al or Cr(VI) stress.	Long PR under ammonium or high Pi conditions. Decreased growth under Fe stress	Short	Less affected	[67,75,75,115,121,123,142,175,177,194,217,257,265]
	aux1-T	*LoF*			Less sensitive to low and high ABA levels							[66]
	aux1-22	*LoF*							Long PR under low B			[244]
	iar3	*LoF*	Long									[125]
	iar4-7 and iar4-8	*LoF*		More affected by salt								[301]

Table S2. Primary root (PR) phenotype of loss or gain of function mutants of genes that participate in hormone homeostasis compared to WT under different abiotic stresses (continuation)

Hormone	Mutant name	Mutant	Type of abiotic stress									References
			Osmotic	Salinity	ABA application	Cold	Oxidative	Heavy metal	Nutrient deficiency	High temperature	Alkalinity	
CK	*ipt3*	*LoF*							Short PR under NO3 supply			[205]
	ipt5	*LoF*										
	IPT3	OE							Reduced growth under K-deficient and K-sufficient conditions			[238]
	ipt3 ipt7	*LoF*						Long PR under Al stress				[176]
	ipt5 ipt7	*LoF*										[176]
	ipt1 ipt3 ipt5 ipt7	*LoF*		More growth					Insensitive to K deficiency			[176,238]
	IPT8	*OE*		Inhibited growth								[130]
	cre1 ahk2	*LoF*						Long PR under Al stress				[176]
	ahk2	*LoF*	Short PR under normal water potential (-0.25 MPa)	More inhibition under 75 or 150 mM salt				Long PR under Al stress	Not affected by K-starved conditions			[112,176,238]
	ahk3	*LoF*	Long PR under low (-1.2 MPa) and moderate water (-0.7 MPa) potential	More inhibition under 150 mM salt					Not affected by K-starved conditions			[112,238]
	ahk4 (cre1-12)	*LoF*	Long PR under normal water potential					Long PR under Al stress	Less sensitive to CK under P availability. Less affected by K-starved conditions			[112,176,224]
	ahk2 ahk3	*LoF*	Long PR under moderate water potential	More inhibition under 75 and 150 mM salt	Short				Short PR under NO3 constant supply. Not affected by K-starved conditions . Short PR under B deficiency			[112,148,149, 205,238,247]
	ahk2 ahk4	*LoF*	More inhibition under low and moderate water potential	More inhibition under 75 mM salt stress					Short PR under NO3 constant supply. Less affected by K-starved conditions			[112,205]

Table S2. Primary root (PR) phenotype of loss or gain of function mutants of genes that participate in hormone homeostasis compared to WT under different abiotic stresses (continuation)

Hormone	Mutant name	Mutant	Type of abiotic stress									References
			Osmotic	Salinity	ABA application	Cold	Oxidative	Heavy metal	Nutrient deficiency	High temperature	Alkalinity	
CK	*ahk3 ahk4*	*LoF*	More inhibition under low water potential						Short PR under NO3 constant supply. Not affected by K-starved conditions			[112,205,238]
	arr1 arr11	*LoF*			Short							[148,149]
	arr1 arr12	*LoF*			Short				Short PR under B deficiency			[148,149,247]
	arr1 arr10 arr12	*LoF*						Long PR under Al stress	Short PR under B deficiency			[176,247]
	arr1 arr11 arr12	*LoF*			Short							[148]
	ARR1	*OE*						Short PR under Al stress				[176]
	ARR12	*OE*						Short PR under Al stress				[176]
	ARR5	*OE*			Short							[148]
	ckx3-1	*LoF*						Short PR under Al stress				[176]
	CKX	*OE*	Long									[126,127,129]
	ugt76c2	*LoF*			Long							[147]
	UGT76C2	*OE*			Short							[147]
GA	*ga1-3*	*LoF*					Less afected by DPI					[165]
	gai	*GoF*							Short PR under low Pi			[226]
	ga1-t	*LoF*							Short PR under low Pi;			[226]
	gai-t6 rga-24	*LoF*				Long						[132]
	sly1-10	*LoF*							Short PR under low Pi			[226]
	gai-t6 rga-t2 rgl1-1 rgl2-1	*LoF*		Long	Less affected	Long			Long PR under low Pi			[117,132,226]

Table S2. Primary root (PR) phenotype of loss or gain of function mutants of genes that participate in hormone homeostasis compared to WT under different abiotic stresses (continuation)

Hormone	Mutant name	Mutant	Type of abiotic stress									References
			Osmotic	Salinity	ABA application	Cold	Oxidative	Heavy metal	Nutrient deficiency	High temperature	Alkalinity	
GA	*ga1-3 gai-t6 rga-t2 rgl1-1 rgl2-1*	*LoF*					More sensitive to DPI		Long PR under low Pi			[165,226]
	gai-t6 rga-t2 rgl1-1 rgl2-1 rgl3-4	*LoF*							Less inhibition under Fe-deficiency			[254]
BR	*det2-9*	*LoF*					Long PR under SOD or DMTU					[166]
	bin2-1	*GoF*			Hypersensitive to ABA							[151]
	bin2-3 bil1 bil2	*LoF*			Less sensitive to ABA							[151]
	bki1-1	*LoF*							Long PR under low Fe			[227]
	bri1	*LoF*	Short	Delayed growth				Less sensitive to Cd	Short PR under low Fe.	Long		[118,134,186,227,255,261]
	bri1-9	*LoFb*			More sensitive to ABA							[152]
	bri1-301	*LoF*							Short PR under B-deficiency			[248]
	bzr1-D	*GoF*		Promoted growth					Not growth inhibition under Low Pi levels. Long PR under low Fe.	Short		[118,227,255,261]
	pUBQ10-bes1-D	*GoF*							Not growth inhibition under Low Pi levels			[228]
	bes1-D	*GoF*							Long PR under B-deficiency	Short		[248,255]
	bri1 brl1brl3	*LoF*	Short									[134]
	bri1-30bak1-3bri1-1bri3-1	*LoF*	Inhibited growth									[134]
	bsk3	LoF							Short PR under low N			[216]
	bsk3 bks4	LoF							Short PR under under high or low N			[216]
	bsk3 bsk4 bsk7	LoF										
	bsk3 bsk4 bsk8	LoF										
	bsk3bsk4bsk7bsk8	LoF										
ABA	*aba1*	*LoF*								Short		[262]
	aba2	*LoF*						Long PR in the co-treatment of Cu and ABA		Short		[201,262]
	aba3	*LoF*								Short		[262]
	perk4-1	*LoF*					Long					[169]
	abi1	*GoF*		Long	Long					Short		[75,117,262]
	abi2	*GoF*			Long					Short		[75,262]
	abi1td	*LoF*			Short							[139]
	abi2td	*LoF*			Short							[139]
	hai1-2 aip1-1	*LoF*			Short							
	hai1-2 hai3-1	*LoF*			Short							
	hai1-2 aip1-1 hai3-1	*LoF*			Short							

Table S2. Primary root (PR) phenotype of loss or gain of function mutants of genes that participate in hormone homeostasis compared to WT under different abiotic stresses (continuation)

Hormone	Mutant name	Mutant	Type of abiotic stress									References
			Osmotic	Salinity	ABA application	Cold	Oxidative	Heavy metal	Nutrient deficiency	High temperature	Alkalinity	
ABA	*hab1-1 abi1-2*	*LoF*						Short PR in the co-treatment of Cu with ABA				[201]
	abi1 abi3 hab1	*LoF*			Long							[146]
Ethylene	*acs1–1 acs2–1 acs4–1 acs5–2 acs6–1 acs7–1*	*LoF*			Long							[146]
	acs2–1 acs4–1 acs5–2 acs6–1 acs7–1 acs9–1	*LoF*			Long							[146]
	acs1–1 acs2–1 acs4–1 acs5–2 acs6–1 acs7–1 acs9–1	*LoF*			Long							[146]
	eto1-1	*LoF*			Short. Long PR under ABA-AVG treatment			Short PR under Al or Cr(VI) stress	Short PR under low or high P Long PR in response to Fe		Hypersensitive	[75,135,146,195,217,234,235,265]
	eto1-2	*LoF*						More inhibition under Al and NAA co-treatment				[175]
	eto2-1	*GoF*							More growth in response to Fe			[251]
	hsp3-1	*LoF*							Short PR under under high or low Pi.			[235]
	hsp3-2	*LoF*							Short PR under under high or low Pi			[235]
	ein2-1	*LoF*			Long			Long PR under Al or Cr(VI) stress. More sensitive to Pb	Not growth repression under low K	Short	Long	[67,75,135,176,179,195,202,243,262,265]
	ein2-5	*LoF*	Short	Short	Long PR under ABA-AVG treatment		Long PR under MV treatment					[135,146,146, 166,177]
	ein3-1	*LoF*									Long	[265]
	ein4-1	*LoF*						Long PR under Cd treatment				[187]
	ein3-1 eil1-1	*LoF*					Long PR under MV treatment	Long PR under Al or Cd stress				[166,176,177, 179,187,188]
	EIL1	*OE*						More growth inhibition under Cd stress				[187]
	EIN3	*OE*						More growth inhibition under Cd stress				[187]
	etr1-1	*LoF*			Long PR under ABA-AVG treatment				Not growth repression under low K			[146,243]
	etr1-3	*LoF*						Long PR under Al or Cr(VI) stress	Reduced growth in response to Fe		Long	[177,195,251,265]

Table S2. Primary root (PR) phenotype of loss or gain of function mutants of genes that participate in hormone homeostasis compared to WT under different abiotic stresses (continuation)

Hormone	Mutant name	Mutant	Type of abiotic stress									References
			Osmotic	Salinity	ABA application	Cold	Oxidative	Heavy metal	Nutrient deficiency	High temperature	Alkalinity	
Ethylene	*ERF1*	*OE*		Long								[136]
	ctr1	*LoF*						More inhibition under Cd stress	Short PR under low or high Pi. Long PR in response to Fe			[187,217,234,243,251]
	ebf1-1	*LoF*						More inhibition under Cd stress				[187]
	EBF1	*OE*						Long PR under Cd treatment				[187]
JA	*opr3*	*LoF*							Long PR under P deficiency.			[236]
	aos	*LoF*						Long PR under Al stress. Short under Cd treatment				[180,189]
	myc2-2	*LoF*						Long PR under Al stress				[180]
	myc2/3/4	*LoF*		Long								[118,137]
	jai3-1	*LoF*		Long								[118,137]
	coi1-2	*LoF*		Long				Long PR under Al stress				[118,137,180]
SA	*sid2-2*	*LoF*							Less PR growth under the cotreatment: SA and high or low N			[210]
	nahG	*OE*						Long PR under Cd stress	Less PR growth under the cotreatment: SA and high or low N.	Short		[190,210,262]
	npr1-1	*LoF*						Less inhibition under Al stress				[179]
SL	*max2-1*	*LoF*							Less reduction of PR length under low Pi.			[237]
CROSSTALK	*ein2 tir1*	*LoF*			Inhibited							[75]
	ein2 aux1	*LoF*			Less inhibition							[75]
	eto1-1 aux1-7	*LoF*									Long	[265]
	ein2-1 npr1-1	*LoF*						More Inhibition under Al stress				[179]
	arf7arf19 coi1-2	*LoF*						Long PR under Al stress				[176]
